# Development and in vivo evaluation of a SARS-CoV-2 inactivated vaccine using high hydrostatic pressure

**DOI:** 10.1038/s41541-025-01136-7

**Published:** 2025-04-25

**Authors:** Martina Brandolini, Pietro Rocculi, Michele Morbarigazzi, Alessandra Mistral De Pascali, Giorgio Dirani, Silvia Zannoli, Davide Lelli, Antonio Lavazza, Francesca Battioni, Laura Grumiro, Simona Semprini, Massimiliano Guerra, Giulia Gatti, Laura Dionisi, Ludovica Ingletto, Claudia Colosimo, Anna Marzucco, Maria Sofia Montanari, Monica Cricca, Alessandra Scagliarini, Vittorio Sambri

**Affiliations:** 1 Unit of Microbiology, The Greater Romagna Area Hub Laboratory, 47522 Cesena, Italy; 2 Department of Medical and Surgical Sciences (DIMEC), Alma Mater Studiorum, University of Bologna, 40138 Bologna, Italy; 3 Department of Agricultural and Food Sciences (DISTAL), Alma Mater Studiorum, University of Bologna, 47521 Cesena, Italy; 4 HPP Italia, 43029 Traversetolo, Italy; 5 Istituto Zooprofilattico Sperimentale della Lombardia e dell’Emilia-Romagna “Bruno Ubertini” (IZSLER), 25124 Brescia, Italy

**Keywords:** Inactivated vaccines, SARS-CoV-2

## Abstract

Developing low-cost vaccine production strategies is crucial to achieving global health equity and mitigating the spread and impact of disease outbreaks. High hydrostatic pressure (HHP) technology is a widely used technology employed in the food industry for long-term preservation. This project aims at validating HHP as a cost-effective method for the production of highly immunogenic thermal stable whole-virus SARS-CoV-2 vaccines. Structural studies on HHP-inactivated viruses demonstrated pressure-dependent effects, with higher pressures (500–600 MPa) destabilizing viral morphology. Immunogenicity assessments, in animal models, revealed that 500 MPa treatment elicited the most robust humoral and cellular immune responses, outperforming heat inactivation. Additionally, HHP-inactivated viral preparation retained thermostability for 30 days at 4 °C, reducing cold-chain dependencies and enabling vaccine distribution also in low-resource settings. With its rapid, cost-effective, and scalable production process, HHP presents a transformative, equitable solution for global vaccine development, particularly for emerging pathogens.

## Introduction

Vaccines are one of the most powerful tools in medicine and public health, transforming once-devastating and human-threatening diseases into preventable diseases. From the eradication of smallpox to the control of polio, vaccine development is the results of decades, if not centuries, of scientific debate and research to overcome technological challenges.

In recent years, the COVID-19 pandemic has brought the importance of vaccine development into sharp focus. In a matter of months, the urge to produce an effective vaccine against COVID-19 has prompted laboratories around the world to engage in an unprecedentedly rapid vaccine development effort to provide effective prophylactic countermeasures to stem SARS-CoV-2 spread in the population^1,2^. A variety of platforms were used by the licensed vaccines (mRNA, viral vector, protein/peptide, and inactivated virus)^3,4^. Interestingly, new approaches, i.e. mRNA vaccines, which have never been tested against infectious disease before the pandemic, and much older technologies, such as whole inactivated vaccines, coexisted^5^. All vaccines authorized by international drug regulation agencies, have thus far proved to effectively reduce morbidity and mortality^6,7^, with new technologies like mRNA vaccines rapidly moving from laboratories to millions of arms across the globe.

While, technically, many challenges have been successfully overcome, the COVID-19 pandemic has brought to the forefront a historical and unsolved health challenge, vaccine equity, with the “global” distribution of SARS-CoV-2 vaccines marked by stark inequalities^8,9^. While high-income countries (HICs) secured early access to vaccines, many low- and middle-income countries (LMICs) have only marginally benefited from immunization campaigns and faced delays in obtaining sufficient doses for appropriate population coverage. This unequal distribution was due to several factors, including but not limited to, lack of pre-purchase agreements and financial resources, and poor logistics, including insufficient cold chain infrastructure^10^. As of August 2024 (latest update of vaccination data), vaccine coverage in most African nations remains significantly lower than in industrialized countries: only about 20% of the population has received at least one dose of a COVID-19 vaccine, compared to over 70% in high-income countries. This vast disparity is most pronounced where logistical, economic, and political challenges have exacerbated the vaccine distribution gap^11^, with staggering examples from Madagascar and Burundi, where the percentage of the vaccinated population (with at least one dose) is below 10% in the former case and below 1% in the latter^12^. This inequity not only resulted in preventable deaths but also prolonged the pandemic, as unvaccinated populations in one part of the world allowed the virus to continue spreading and mutating^13–15^.

Developing production strategies for low-cost and thermal-stable vaccine is essential to meet growing global demand, especially in LMICs for achieving health equity. Producing low-cost vaccine production is not only a moral imperative but also a strategic necessity^16^. Increased vaccine accessibility promotes health security and stability by reducing the risk of disease spread, which finally benefits both LMICs and HICs by preventing global diseases spread^17,18^.

High hydrostatic pressure (HHP) processing has emerged in the food industry as a valuable non-thermal food preservation technique used for microbial inactivation in food products^19–21^. HPP (also known as HPP, High Pressure Processing) involves the application of hydrostatic pressures typically ranging from 100–800 MPa at refrigeration or mild process temperatures (<45 °C) to food products, often for seconds or minutes^22–24^. In contrast to thermal pasteurization, HHP minimizes heat-induced changes, offering a promising solution for preserving heat-sensitive components^25–28^. The use of HHP for the inactivation of viruses has been the subject of published studies for a range of foodborne viruses investigating the factors affecting HHP efficacy, including temperature, pH, strain diversity, and combined treatments (e.g., marination), collectively demonstrating HHP’s potential to enhance food safety, particularly in high-risk foods, by effectively reducing viral contamination without compromising food quality^29–34^.

Whilst the majority of studies on HHP focused on the inactivation of foodborne viruses, HHP has been explored as an innovative approach for vaccine production, demonstrating its ability to inactivate viruses whilst preserving antigenic structures crucial for immune recognition^35,36^. This has been investigated in several studies targeting a range of pathogenic viruses. For instance, Barroso et al.^35^ successfully inactivated avian influenza viruses using HHP, paving the way for vaccine development against zoonotic threats. Similarly, de Souza et al.^36^ explored the effects of HHP on the immune recognition of antigens from porcine parvovirus, highlighting its potential for veterinary vaccines. The seminal work of Shearer and Kniel^37^ and the subsequent studies by Silva et al.^38^ laid the foundation for the understanding of HHP inactivation across a wide spectrum of human and animal viruses. These findings collectively underscore the potential of HHP not only for food safety but also as a platform for developing effective vaccines against emerging and re-emerging viral threats, hence suggesting that this technology has promising potential to drive the next generation of vaccines. Moreover, drawing from its application in the food industry, where HHP enables effective long-term preservation of product quality at refrigeration temperatures, HHP technology offers the potential to produce vaccines that remain stable at refrigeration or ambient temperatures, reducing or eliminating the reliance on cold chain logistics for distribution^39^.

This study was aimed at developing and validating a vaccine production methodology based on the viral inactivation achieved through HHP processing. By validating the process for SARS-CoV-2, this research aims to establish HHP as a versatile and cost-effective process for producing whole virus inactivated vaccines that combine affordability with high immunogenic potential, responding to requirements for marketing authorization of the European Medicine Agency^40^.

## Results

**Fig. 1:**
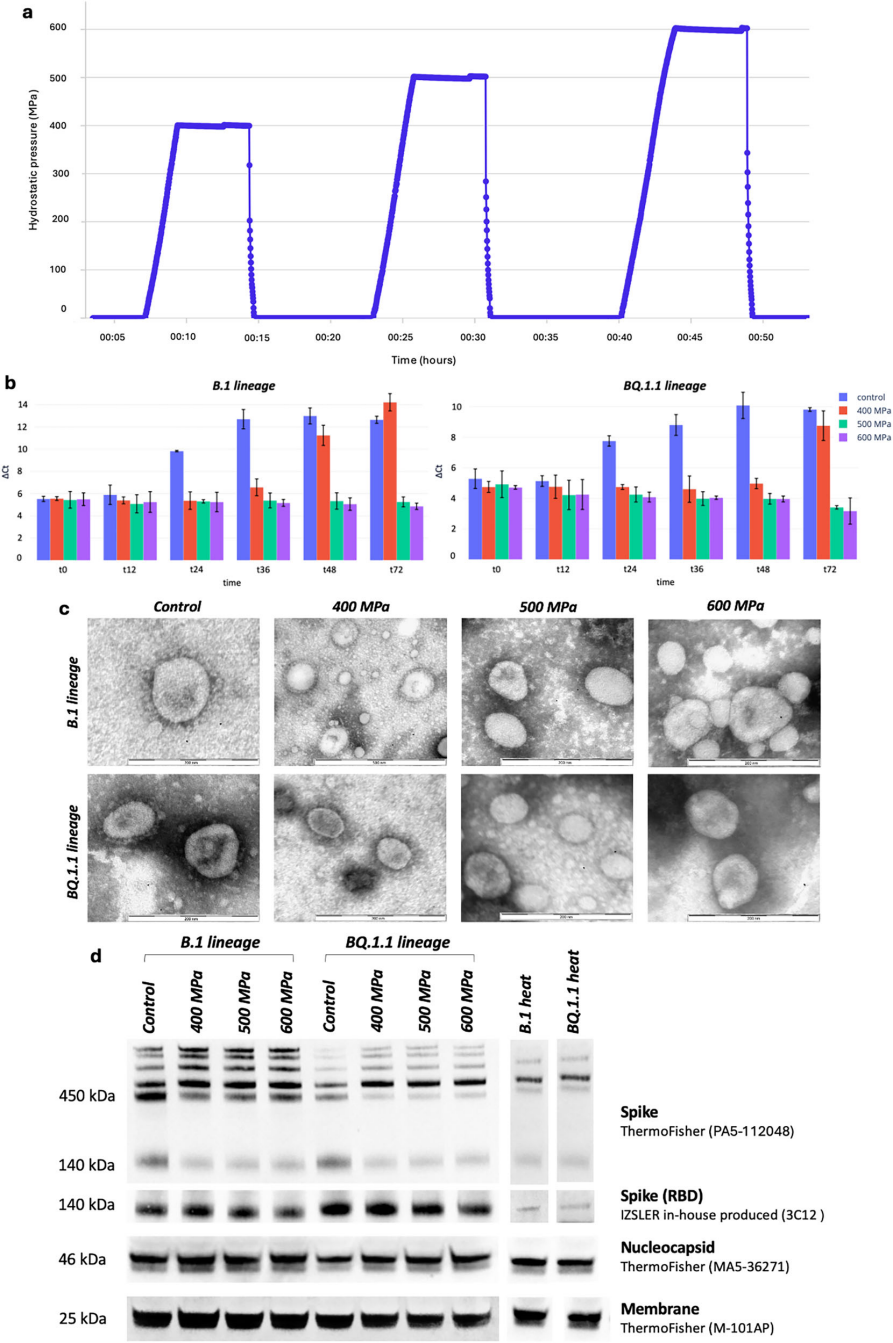
HHP treatment of viral isolates and its effects on viral structure and antigenicity. **a** Pressure profile of high hydrostatic pressure (HHP) treatment applied over time. Each pressure was maintained for a fixed duration, followed by a rapid decompression phase to atmospheric pressure. After each pressurization cycle, the viral suspension subjected to the treatment was removed and replaced with a fresh suspension for the following cycle. The machine was maintained active throughout the entire series of treatments, continuously recording pressure, including during decompression phases when the pressure returned to ambient levels, resulting in the appearance of a sequential pressure profile in the figure, although each cycle was conducted separately. **b** Viral replication dynamics, measured as ΔCt values of Real-Time PCR, for HHP-treated and non-HHP treated B.1 and BQ.1.1 lineages over time. C. nsEM microscopy results for 400–600 MPa-treated SARS-CoV-2 isolates compared to the control (non-HHP-treated). **d** Results of western blot analyses conducted to test the ability of viral proteins to bind polyclonal and monoclonal antibodies after inactivation at high pressures.

Hermetically sealed polyethylene pouches containing viral suspensions were subjected to HHP treatment at 400, 500 and 600 MPa for 5 minutes. The operating pressure conditions were chosen to minimize the total processing time, aiming to develop a potentially high-throughput inactivation system. This approach required operating at relatively high pressures to achieve complete viral inactivation, in contrast to other inactivation protocols described in the literature^35,36^, which employ lower pressures but require significantly longer treatment durations, on the order of hours rather than minutes. Each pressure condition was applied as an independent treatment cycle. After each pressurization cycle, the viral suspension subjected to that specific pressure was removed, and a fresh, untreated viral stock was introduced for the next cycle. This ensured that each viral stock was exposed to only one pressure condition, preserving the integrity of independent treatments. Pressure inside the chamber was continuously recorded throughout the entire series of treatments, including during decompression phases when the pressure returned to ambient levels, hence producing a sequential pressure-time profile (Fig. 1a). This methodological choice streamlined the workflow while maintaining precise control over pressure conditions.

### Inactivation assessment

After HHP treatment, conducted as reported in Fig. 1a, virus infectivity reduction was assessed by endpoint titration. Treatment at 400 MPa resulted in a significant reduction of 2.9 and 3.9 log10 for B.1 and BQ.1.1 compared to the non-HHP-treated control. At 500 MPa and 600 MPa, no infectious virus was detected, suggesting complete viral inactivation for both lineages. Complete inactivation was further tested by culturing the putatively inactivated viral stock on Vero E6 cell culture; viral replication was evaluated by qRT-PCR. 400 MPa treatment temporarily suppressed viral replication, but was insufficient to maintain long-term inhibition, allowing a significant rebound in viral replication for both lineages at 48 h post-infection. At 500 and 600 MPa, viral replication was consistently suppressed, and viral load declined progressively from 0 to 72 h post-infection, indicating that both pressures exerted a stronger inhibitory effect on viral replication, leading to complete abrogation (Fig. 1b).

### Negative Staining Electron Microscopy (nsEM)

The analysis revealed distinct morphological effects depending on the applied pressure, providing insights into the impact of HHP on viral ultrastructure and potential mechanisms of viral inactivation.

At 400 MPa, nsEM images demonstrated that the overall morphology of the viral particles was fully preserved. The virions exhibited a typical coronavirus structure, characterized by spherical particles with clear surface spikes (S proteins) protruding from the envelope (Fig. 1c, Control). This indicated that treatment at 400 MPa, while effective in reducing (but non abrogating) infectivity, did not induce significant structural damage to the virus (Fig. 1c, 400 MPa). In contrast, viruses treated at 500 MPa displayed a slight alteration in their morphology. While viral particles remained visible and could still be identified as coronaviruses, there were changes in their structural appearance, including a less defined or partially distorted spike arrangement (Fig. 1c, 500 MPa). However, the generally spherical shape of the virions was retained. This suggests that HHP at 500 MPa began to compromise the integrity of surface structures, potentially affecting the ability of the virus to engage with host receptors, which may contribute to its inactivation. At 600 MPa, the morphological changes were more pronounced and indicative of significant structural disruption (Fig. 1c, 600 MPa). nsEM images showed a substantial reduction in the number of identifiable viral particles. Among the particles that remained visible, the surface spikes were almost entirely absent, resulting in virions with a smooth appearance. This loss of spike proteins, which are critical for viral attachment and entry into host cells, likely explains the observed inactivation at this pressure level. In addition, the overall structure of the virions appeared to be severly compromised, with many particles showing irregular or collapsed shapes, many forming aggregates, further supporting the conclusion that 600 MPa exerts a profound destabilizing effect on the virus.

### Western blot

Western blot analysis of the spike protein of viruses treated with HHP at 400 MPa, 500 MPa, and 600 MPa revealed interesting changes in the protein profile. For all pressure levels tested, a reduction in the signal corresponding to both monomeric ( ~ 140 kDa) and trimeric ( ~ 450 kDa) forms of the spike protein was observed. Reduction was more pronounced for higher pressures, although the difference was non-significant, suggesting that high-pressure treatment may have led to some level of structural destabilization or dissociation of the spike protein. In contrast to the reduction of the monomeric and trimeric spike signals, an interesting finding emerged regarding higher molecular weight bands ( > 450 kDa). These bands, which were less represented in untreated virus samples, showed a noticeable increase in intensity as the applied pressure increased. It is likely that the application of high hydrostatic pressure could have promoted the aggregation of spike proteins or other viral components, resulting in the formation of larger complexes or aggregates. These high molecular weight bands were particularly prominent at higher pressures, with the 600 MPa treatment leading to the most intense signals, suggesting that the extent of macromolecular aggregation is pressure dependent. Western blot analysis was also conducted on other structural proteins of the virus, specifically the M and the N proteins. The results revealed no substantial differences between the treated samples and the untreated control, nor were there any discernible changes between the different pressure treatments in terms of signal intensity and band pattern.

Heat-inactivated viral propagates, subsequently used as controls for mouse immunizations, were also tested, exhibiting a stark contrast to the pressure-treated samples. Notably, the signal corresponding to the spike protein was completely absent in the heat-inactivated virus, indicating a complete loss of this critical viral protein upon heat treatment. This loss suggests that heat inactivation induces more severe structural damage to the spike protein compared to HHP treatment. In contrast, the M and N proteins in the heat-inactivated virus showed only a partial reduction in their respective signals, suggesting that while heat treatment affected these proteins to some extent, it did not lead to their complete degradation (Fig. 1d).

### In vivo Immunogenicity: B cell responses

**Fig. 2:**
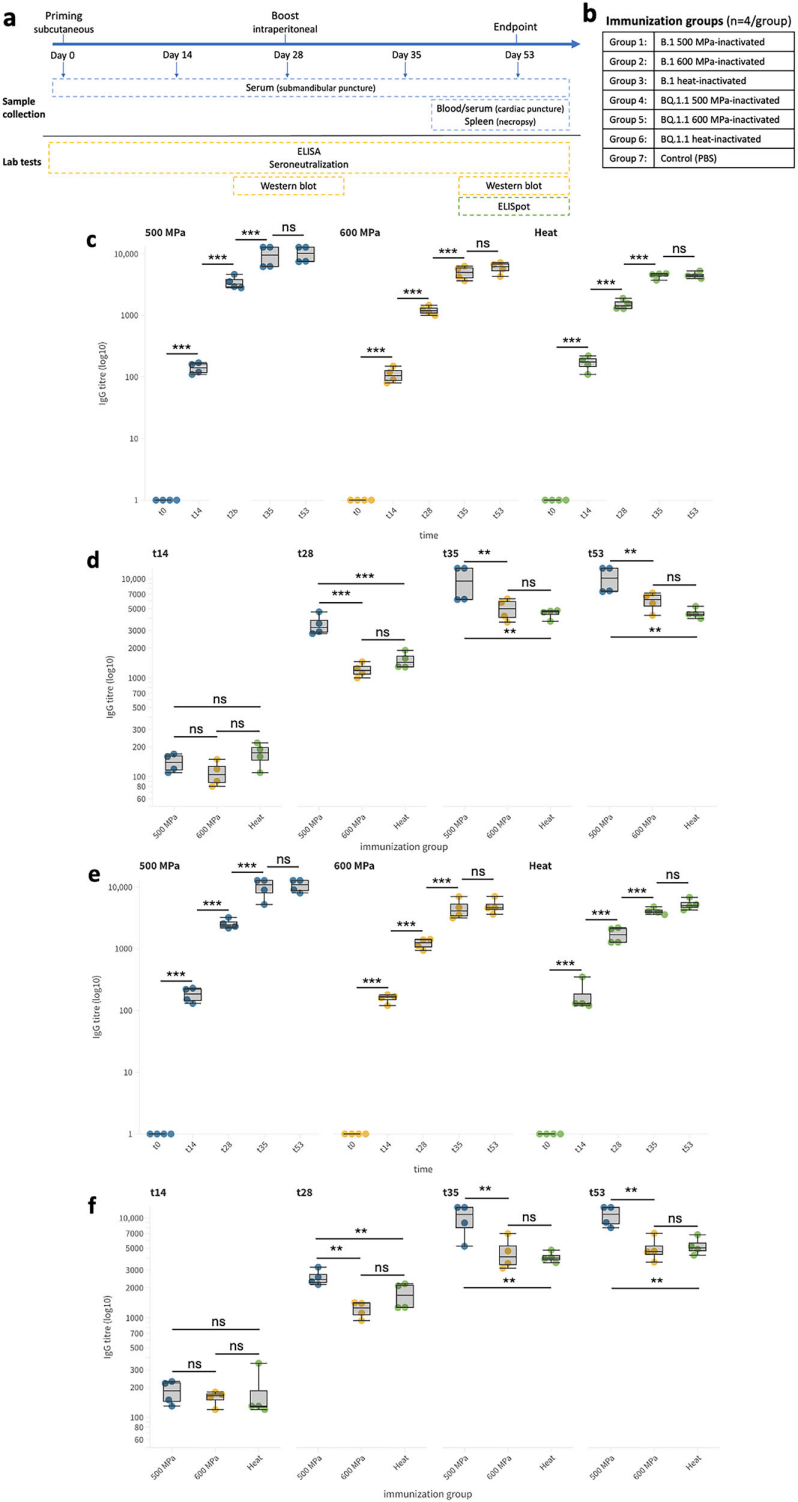
HHP-inactivated SARS-CoV-2 in vivo safety and immunogenicity testing and activation of specific humoral responses. **a** Experimental timeline and protocol for mouse immunization, including priming (day 0), boosting (day 28), biological samples collection, and endpoint (day 53). **b** Immunization groups. **c**, **e** IgG titers measured via ELISA for B.1 (**c**) and BQ.1.1 (**e**) lineages. **d**, **f** Comparison of IgG titers at distinct time points for B.1 (**d**) and BQ.1.1 (**f**) lineages. Statistically significant differences are indicated: ***p < 0.001, **p < 0.05, ns = not significant.

Immunogenicity of HHP-inactivated SARS-CoV-2 was tested in Swiss CD1 mice subcutaneously vaccinated and intraperitoneally boosted at day 28 with 10 µg of inactivated virus (Fig. 2a, b). The levels of anti-SARS-CoV-2 IgG responses against whole virus were evaluated at days 14, 28, 35 and 53 after first immunization. The seroconversion rate was 100% at 14 days after immunization in HHP-immunized groups as well as in heat-immunized groups and antibody responses steadily but consistently increased until day 28. After the booster dose, antibody titers in both HHP-inactivated immunization groups increased consistently and peaked at day 35 (7 days after second immunization). In contrast, for heat-inactivated immunization group peak titer was reached on day 53. The One Way ANOVA test indicated a significant difference in the antibody titer among the different time points for both tested viral lineages and for all immunization groups (B.1 lineage: *F* = 15.2, *p* = 2.19E-4 for 500 MPa-inactivated; *F* = 39.4, *p* = 1.74E-6 for 600 MPa-inactivated; *F* = 118.8, *p* = 3.45E-09 for heat-inactivated; BQ.1.1 lineage: *F* = 48.8, *p* = 5.37E-07 for heat-inactivated; *F* = 22.31, *p* = 3.37E-5 for 500 MPa-inactivated; *F* = 17.90, *p* = 9.99E-5 for 600 MPa-inactivated). The post-hoc Tukey’s HSD test for pairwise comparisons highlighted a consistent and statistically significant increase from day 0 to 14, 14 to 28 and 28 to 35 in all groups. Afterward, antibody titers plateaued in all experimental groups, with only slight and non-significant increases or decreases at day 53 (Fig. 2c for B.1 lineage and Fig. 2e for BQ.1.1 lineage). No SARS-CoV-2 specific antibody response was detected in the control group.

When comparing the three groups at each time point, the 500 MPa HHP-treated group consistently showed the highest antibody titers at t28 (B.1: *F* = 21.81, *p* = 3.54E-4; BQ.1.1: *F* = 10.38, *p* = 4.60E-3), t35 (B.1: *F* = 4.83, *p* = 3.76E-2, BQ.1.1: *F* = 7.72, *p* = 1.11E-2), and t53 (B.1: *F* = 9.08, *p* = 6.95E-3; BQ.1.1: *F* = 12.74, *p* = 2.37E-3). At all three time points, the titers for the 500 MPa group were significantly higher than those of the heat-inactivated and 600 MPa groups, indicating a stronger humoral immune response. Furthermore, the antibody titers in the 500 MPa group remained high and sustained throughout the time course, indicating a robust immune response. In contrast, the heat-inactivated group showed a slower increase and lower overall antibody titers and lower titers compared to the 500 MPa group. The 600 MPa HHP-treated group showed a general increase in antibody titers over time, consistently exhibiting lower titers compared to the 500 MPa group. No statistically significant differences were detected between the 600 MPa group and the heat-inactivated group (Fig. 2d for B.1 lineage and Fig. 2f for BQ.1.1 lineage).

The immune response elicited by HHP-inactivated or heat-inactivated viruses was comprehensively evaluated through Western blot analysis at t28 and t53 to assess the specific antibody profiles induced by immunization and evaluate the progression and maturation of the immune response over time.

**Fig. 3:**
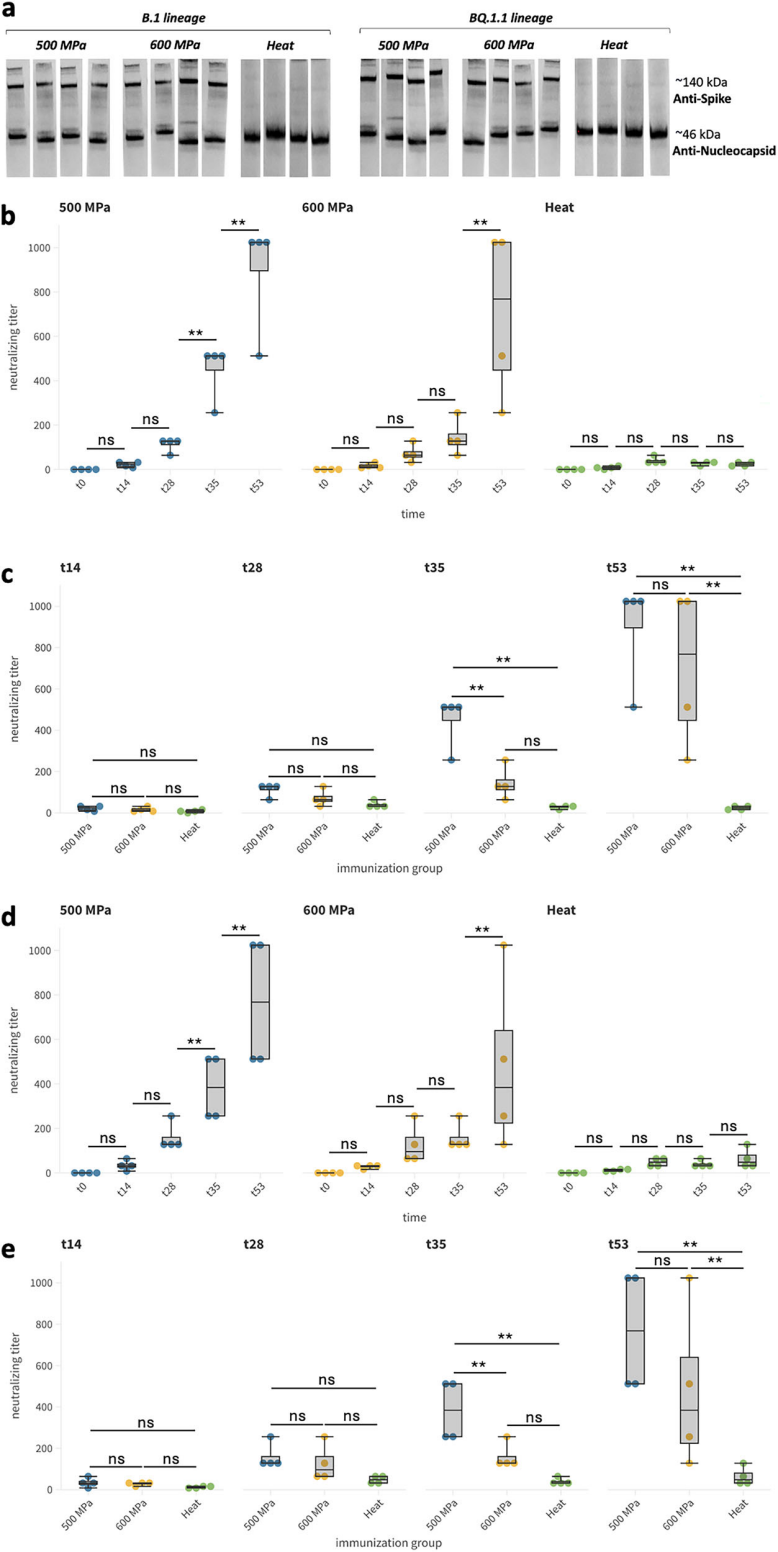
Induction of neutralizing antibody response in HHP-inactivated SARS-CoV-2 vaccinee. **a** Western blot analysis of sera from mice immunized with B.1 and BQ.1.1 lineage viruses inactivated by 500 MPa, 600 MPa HHP, or heat. **b**–**e** Neutralizing antibody titers in immunized mice measured at different time points. Images B and D show longitudinal neutralization titers for B.1 (**b**) and BQ.1.1 (**d**) lineages. Images (**c**, **d**) show the comparison of neutralizing titers between immunization groups. Statistical significance: ***p < 0.001, **p < 0.05, ns not significant.

Results revealed marked differences between the various groups of mice immunized with different viral inactivation treatments. Specifically, mice that were immunized with the virus treated with HHP at 500 MPa and 600 MPa exhibited a broader antibody response. These mice produced antibodies not only against the S protein but also against the N protein. This was evident from the detection of two distinct bands in the Western blot corresponding to the expected molecular weights of the S and N proteins. The detection of these bands at both t28 and t53 indicates that the immune response was strong and sustained against both proteins and persisted over time. In contrast, mice immunized with the heat-inactivated virus presented a markedly different antibody profile. These animals developed antibodies that specifically targeted the N protein at both time points. However, no significant antibody response against the S protein was observed. Neither anti-S nor anti-N antibodies were detected in the control group (Fig. 3a).

Neutralizing activity of humoral response was assessed on days 14, 28, 35, and 53 post-immunization, allowing for both intragroup (within the same group at different time points) and intergroup (comparison across groups at the same time point) evaluations.

A neutralizing antibody response was detectable in all groups at 14 days after immunization. The 500 MPa group exhibited a clear increase in neutralizing antibody titers over time. Like the 500 MPa group, the 600 MPa group displayed increasing titers over time, albeit with a slightly less consistent trend and slightly weaker responses compared to the 500 MPa group. For both groups and both tested variants, the Kruskal-Wallis test with Dunn’s post-hoc test with Bonferroni correction highlighted a significant increase in the neutralizing titer over time (B.1 lineage: χ2 = 14.03, p = 0.003 for 500-MPa inactivated; χ2 = 13.08, p = 0.004 for 600 MPa-inactivated; BQ.1.1 lineage: χ2 = 13.52, p = 0.004 for 500-MPa inactivated; χ2 = 11.22, p = 0.011 for 600-MPa inactivated). The heat-inactivated group exhibited the weakest antibody response, with a poor initial immune response, and a slight improvement over time, with no substantial increase, suggesting that heat inactivation was less effective at eliciting a robust or sustained neutralizing antibody response compared to HHP treatments (Fig. 3b for B.1 lineage and Fig. 3d for BQ.1.1 lineage). No effective neutralizing response was detected in the control group.

Regarding intergroup comparisons, as early as t14, all groups displayed relatively low titers, with minor and non-significant differences that may reflect variability in individual immune responses. By t28, the 500 MPa group exhibited higher titers compared to the 600 MPa group and the heat-inactivated group. At t35 differences became more pronounced: the 500 MPa group showed the highest titers, followed by the 600 MPa group. Indeed, titers did not increase for the heat-inactivated group compared to the previous timepoint. These results indicate that both HHP treatments outperformed heat inactivation in driving a strong neutralizing antibody response, with the 500 MPa group demonstrating the most robust response (*χ2* = 9.89, *p* = 0.007 for B.1 and *χ2* = 9.71, *p* = 0.008 for BQ.1.1). By the final time point (t53), the 500 MPa group maintained the highest titers. The 600 MPa group followed closely. In the heat-inactivated group titers plateaued with a statistically evident difference from both HHP-inactivated groups: *χ2* = 8.29, *p* = 0.016 for B.1 and *χ2* = 7.9, *p* = 0.019 for BQ.1.1 (Fig. 3c for B.1 lineage and Fig. 3e for BQ.1.1 lineage).

### In vivo immunogenicity: T-cell responses

**Fig. 4:**
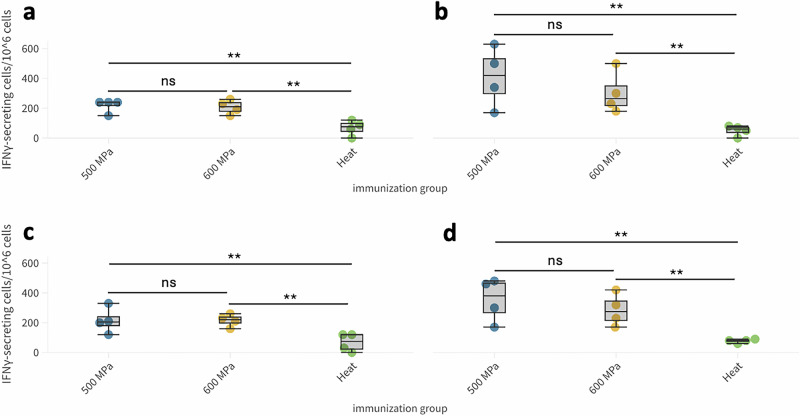
Induction of T-cell response in HHP-inactivated SARS-CoV-2 vaccinee. **a**, **c** IFN-γ ELISpot results for PBMCs from mice immunized with B.1 (**a**) and BQ.1.1 (**c**) lineage viruses inactivated by 500 MPa, 600 MPa HHP, or heat. **b**, **d** IFN-γ ELISpot results for splenocytes from mice immunized with B.1 (**b**) and BQ.1.1 (**d**) lineage viruses. Statistical significance: **p < 0.05, ns = not significant.

T-cell responses were evaluated on day 53 from PBMC and splenocytes. The 500 MPa group showed robust T-cell responses in both whole blood and spleen samples. The 600 MPa group also exhibited a notable T-cell response, though slightly less consistent than the 500 MPa group. These findings highlighted the effectiveness of the 500 MPa inactivation method in preserving viral antigens capable of eliciting a strong cellular immune response, particularly in the spleen, which serves as a primary site of immune activation. Inactivation at 600 MPa, albeit effective, may be slightly less reliable in consistently stimulating T-cell activation compared to the 500 MPa method. Nonetheless, the overall cellular immune response in this group revealed to be higher than that observed in the heat-inactivated group, which displayed the weakest T-cell-mediated immune responses among the three groups, with most individuals showing minimal responses, suggesting that the heat inactivation process likely denatures or alters viral antigens, thereby reducing their immunogenicity and ability to effectively stimulate T-cell responses. This finding is consistent with observations from other immune parameters, where heat inactivation resulted in weaker immune responses compared to HHP methods. Statistical analysis carried out with One Way ANOVA test highlighted a significant difference between 500-MPa inactivated and heat-inactivated groups and between 600-MPa inactivated and heat-inactivated groups on both PBMC, reported in Fig. 4a and c for B.1 and BQ.1.1 lineage, respectively, and splenocytes, reported in Fig. 4b and d for B.1 and BQ.1.1 lineage, respectively, (B.1 lineage: *F* = 12.1585, *p* = 0.0027 for PBMC and *F* = 6.6474, *p* = 0.01687 on splenocytes; BQ.1.1 lineage: *F* = 6.7526, *p* = 0.01617 for PBMC and *F* = 7.3906, *p* = 0.01262 for splenocytes). No significant difference was highlighted between the two HHP treatments (Fig. 4). No SARS-CoV-2 specific T-cell response was detected in the control group.

### Thermostability assessment

**Fig. 5:**
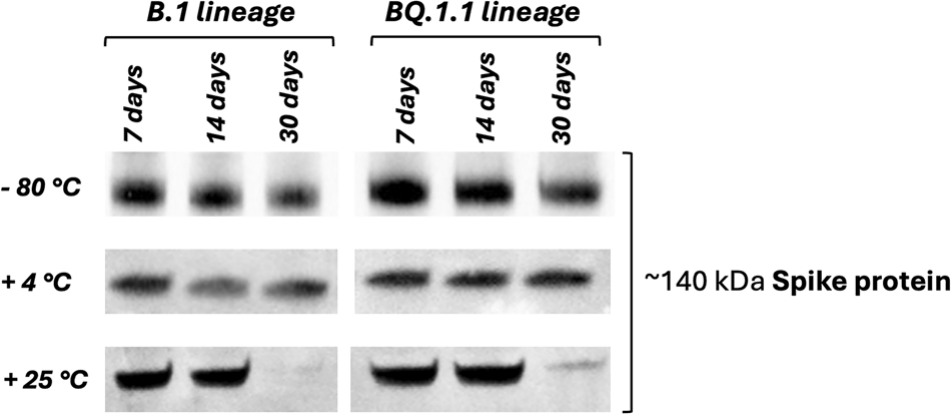
Western blot analysis of the Spike protein signal from SARS-CoV-2 B.1 and BQ.1.1 variants inactivated at 500 MPa, stored at different temperatures (-80 °C, 4 °C, 25 °C). Vaccine candidate aliquots stored under different conditions were analyzed over various time points (7, 14, and 30 days).

The thermostability of 500 MPa-inactivated and purified preparation was assessed to evaluate the preservation of antigenic stability under different storage conditions for different periods of time. The results demonstrated that aliquots stored at 4 °C retained antigenic stability across all evaluated time points, showing almost undetectable loss of antigenic integrity. In contrast, the aliquots stored at room temperature exhibited stability only up to 14 days, with a noticeable decline in antigenic quality observed by day 30. These findings underscored the robustness of the HHP inactivation in preserving the antigenic properties of virions, making refrigeration a viable alternative to freezing for the storage of HHP-inactivated viral preparations (Fig. 5).

## Discussion

The inequality in vaccine distribution experienced during the COVID-19 pandemic, between high- and low-income countries, has highlighted not only the moral imperative of vaccine access but also the practical reality that no country is safe from a pandemic unless all countries are, underscoring the need for a more equitable global public health system, where vaccines and other health interventions are accessible to all, regardless of geography or income^41^. The ongoing disparity in vaccine access is a pressing global health challenge that highlights the need for innovative, scalable, and cost-effective vaccine production and distribution strategies. High hydrostatic pressure offers a promising alternative to traditional vaccine production methods, representing an approach with significant potential to address some key barriers to equitable vaccine distribution. Besides technical advantages represented by preserved antigenicity, efficacy across viral variants, thermostability, and rapid processing, HHP offers economic benefits, enabling low-cost, large-scale production while reducing cold-chain dependencies. Socially, HHP may enhance vaccine accessibility, particularly in low-resource settings, due to their low-cost production and improving the requirements for vaccine storage and handling, ensuring equitable distribution and faster response to pandemics. These benefits make HHP a transformative technology for global vaccine development, addressing both health equality and logistical challenges.

Although the majority of HHP studies have concentrated on inactivating foodborne viruses, growing evidence supports its application in vaccine development, showcasing its capability to inactivate viruses while maintaining antigenic structures vital for immune recognition. This innovative approach has been explored across various pathogenic viruses, positioning HHP as a promising platform for vaccine production, providing pivotal contribution to expand the understanding of HHP’s effectiveness across a diverse range of human and animal viruses^35–38^. Building on this body of work, our study further explores HHP potential, situating our research within the existing framework and advancing the application of HHP in vaccine development. By leveraging the groundwork laid by previous studies, we aim to contribute to the evolving field of HHP-based vaccines, which holds significant promise for addressing emerging and re-emerging viral threats.

The present study focuses on the development and validation of a novel method for vaccine production using HHP technology, investigating the effects on viral inactivation, and understanding the structural alterations induced by pressure and their impact on immunogenicity. The research focused on structural and antigenicity analysis of HHP-treated SARS-CoV-2 virus, followed by immune response assessment in an animal model.

The first step in this study was to investigate the pressure-induced morphological changes of HHP-treated viral isolates. Our findings highlighted the pressure-dependent effects of HHP on viral replication and morphology. At 400 MPa, the virus exhibited a partial suppression of infectivity, with only minor alterations in its structural integrity. However, at higher pressures, significant morphological damage was observed, especially at 600 MPa, which led to an almost complete loss of surface spike proteins, a key structural component essential for cell receptor engage and cell infection, providing evidence for a potential mechanism of viral inactivation. Furthermore, Western blot analysis highlighted pressure-induced alterations in the spike protein, including the reduction of both the monomeric and trimeric forms, alongside the formation of high molecular weight aggregates, more abundant at higher pressure and potentially indicative of macromolecular aggregation, a phenomenon that may be triggered by the HHP treatment and may contribute to viral inactivation. Moreover, when compared with heat inactivation, HHP treatment resulted in less overall protein degradation, particularly in the spike protein, suggesting that HHP might offer a less disruptive method of inactivating the virus while maintaining a more stable overall protein structure.

Following the structural analysis, we evaluated the immunogenicity of the HHP-inactivated viruses in an animal model. Our findings demonstrated that the method of inactivation and the applied pressure significantly influenced the immune responses. Treatment at 500 MPa induced a robust and complete immune response, producing the highest and most sustained IgG antibody titers. In contrast, the heat-inactivated virus elicited lower peak antibody titers and a slower immune response, highlighting the efficacy of HHP in generating a more potent humoral immune response. Treatment at 600 MPa, while still effective, resulted in the weakest IgG response, with lower antibody titers across all time points. When analyzing neutralizing antibody responses, both 500 MPa and 600 MPa treatments outperformed the heat-inactivated group. The neutralizing antibody titers in the 500 MPa group were consistently the highest, followed by the 600 MPa group, which demonstrated robust neutralizing capacity, despite exhibiting the lowest overall antibody (IgG) responses. The observed difference in the magnitude and kinetic of the humoral response correlate with antigenic integrity. In fact, 500 MPa HHP treatment, preserved key antigens like the spike protein thus elicited the strongest and most sustained antibody titers. Heat inactivation, on the contrary, and as highlighted by Western blot analysis, caused antigen degradation, leading to weaker responses. Meanwhile, the 600 MPa HHP treatment, though preserving viral structure to some extent, induced a weaker immune response, possibly due to excessive pressure causing subtle structural disruptions that impacted antigenicity. Supporting the findings from the neutralization assays, Western blot analyses performed on sera from immunized mice confirmed the results of the neutralization assay and revealed significant differences in the immune responses elicited by HHP-treated and heat-inactivated viral preparations. The HHP-treated viruses induced a more comprehensive antibody response, targeting both the spike and nucleocapsid proteins, while the heat-inactivated virus generated a response primarily against the nucleocapsid protein. This observation is particularly significant given that the spike protein is the primary inducer of neutralizing antibody responses. The absence of a spike-specific response in the heat-inactivated group likely accounts for the weaker neutralizing antibody titers observed in this group, underscoring the importance of preserving the structural integrity of the spike protein during inactivation to ensure the generation of robust and protective immune responses. These findings highlight the advantages of HHP inactivation in maintaining the antigenic properties of key viral proteins critical for eliciting effective immunity.

In addition to antibody responses, T-cell activation was also assessed. The 500 MPa treatment consistently elicited the strongest and most reliable T-cell responses, particularly in spleen samples. The 600 MPa treatment also triggered T-cell activation, though with more variability. In stark contrast, the heat-inactivated group showed minimal T-cell activation, both in whole blood and spleen, which emphasized the limitations of heat inactivation in eliciting a strong cellular immune response. This broader immune response generated by HHP inactivation was seen as a significant advantage in the context of vaccine development, as it could provide a more comprehensive protective immune response.

In light of the above results, this study provides strong evidence for the potential of HHP as an effective method for viral inactivation for vaccine production, demonstrating its ability to preserve viral antigenicity while eliciting robust immune responses, hence offering a more efficient and less disruptive alternative to traditional heat inactivation methods. Importantly, HHP inactivation demonstrated higher immunogenicity in both humoral and cell-mediated immune responses, with the 500 MPa treatment showing the most promising results.

The thermostability of the HHP-inactivated viruses was also assessed as a critical requisite to improve vaccine handling and storage. The ability to maintain antigenic integrity under various storage conditions is a crucial factor in the practicality of vaccine distribution, particularly in regions with limited access to ultra-cold storage facilities. Our findings demonstrated that HHP-inactivated viruses, when stored at refrigeration temperatures (4 °C), maintained their antigenic stability for at least 30 days. Our results suggest that HHP-inactivated vaccines could be stored under less stringent conditions than those requiring ultra-cold freezing, thereby reducing logistical challenges and costs associated with cold-chain maintenance, which poses significant challenges in low-resource settings.

The study has also demonstrated a key scientific and practical strength of the HHP process: its physical mechanism of action makes it unlikely that genetic variations among viral strains would exert an influence on the efficacy of the inactivation process. This is evidenced by our findings, which yielded comparable results with ancestral B.1 and more heavily mutated BQ.1.1 lineages. Considering this, HHP represents a powerful tool for ensuring an agile and effective vaccine response to the ongoing evolution of viral threats, thereby reducing the time required to develop and deploy vaccines against emerging variants. This feature has significant implications for the development of vaccines, particularly in the context of rapidly mutating RNA viruses such as influenza and coronaviruses, which present significant challenges to traditional vaccine development methods.

Additionally, one of the most significant operational advantages of the HHP method is the markedly brief processing time, compared to other HHP inactivation protocols proposed in literature^35,36^. The high-pressure treatment phase typically lasts only a few minutes and enables the rapid inactivation of large volumes of viral suspension (up to 400–450 liters) within a single batch. The high throughput of the HHP process aligns with the demands of industrial-scale vaccine production and may be particularly advantageous in outbreak scenarios, where speed is crucial to responding to the demand for vaccines in a timely manner, thus mitigating bottlenecks in vaccine production. A further crucial benefit of the HHP process is its low operational cost. The mass production of viral suspensions using HHP is estimated to cost ~0.10–0.30 € per kilogram^27^, which represents a significant reduction in cost compared to traditional production methods. The affordability of HHP-based production could have a transformative impact on the development of cost-effective vaccines that can be accessible to a broader population, particularly in LMICs. This could lead to a reduction in the financial burden on healthcare systems and international aid organizations tasked with vaccine distribution^13,42^.

Lastly, on the social level, the use of the same inactivated virus as the basis for the vaccine could enhance public trust and acceptance, particularly among individuals who harbor concerns about vaccines developed using advanced molecular techniques, such as genetic engineering or mRNA-based platforms. This approach leverages traditional and well-established vaccine production methodologies, which are often perceived as safer and more “natural” by vaccine-hesitant groups. Consequently, it may help overcome resistance rooted in fears of novel technologies, thereby facilitating higher uptake rates and contributing to more effective immunization campaigns, albeit the present corpus of scientific literature does not provide an unambiguous consensus on an overall preference for inactivated vaccines over mRNA vaccines, as vaccine acceptance may be influenced by cultural factors, the availability of the vaccines, public communication, and trust in local health authorities^43,44^.

Despite its immense promise, the implementation of HHP technology for vaccine production is not without challenges and more extensive research is needed to further explore the use of HHP in vaccine production. Although this study focused on SARS-CoV-2 as an effective proof of concept, the principles of HHP inactivation are broadly applicable to other viruses of public (human and animal) health importance, including Influenza, Dengue, West Nile, or African Swine Fever. Notwithstanding the necessity to identify the correct parameters of pressure and exposure times to achieve optimal inactivation while preserving antigenicity, the adaptability of HHP technology nonetheless makes it a particularly attractive and valuable platform in the context of pandemic preparedness, where rapid response capabilities are critical to control the spread of diseases.

In conclusion, the development of HHP technology for vaccine production represents a significant step forward in addressing the global challenges of vaccine access and distribution. By combining cost-effective production methods with enhanced antigenic preservation and thermostability, HHP-based vaccines have the potential to bridge the gap between high- and low-income countries, ensuring more equitable access to life-saving immunizations. While further research and validation are needed to fully realize the potential of this technology, its scalability, adaptability, and cost-effectiveness make it a promising platform for addressing both current and future global health challenges. As the world continues to grapple with the dual burdens of infectious diseases and inequitable healthcare access, innovative approaches such as HHP technology will be critical for building a more inclusive and resilient global health system.

## Materials and methods

### Cells and virus

Vero E6 (ATCC CRL 1585) cell cultures were maintained in Minimum Essential Medium (MEM) supplemented with 10% heat-inactivated fetal bovine serum (FBS), 2 mM L-glutamine, 100 U/mL penicillin, 100 μg/mL streptomycin (EuroClone, Milan, Italy) and incubated at 37 °C in a humidified, 5% CO_2_ atmosphere-enriched chamber^45^. B.1 and BQ.1.1 strains were isolated from SARS-CoV-2 positive nasopharyngeal swabs residual from routine activities and submitted to the Microbiology Unit, Greater Romagna Area Hub Laboratory, Cesena, Italy, for diagnostic purposes, as described previously^46^. Samples were sequenced as part of the project to monitor the prevalence and distribution of SARS-CoV-2 variants in Italy, sponsored by the Italian Institute of Public Health (ISS). Samples were sequenced using CleanPlex SARS-CoV-2 Flex (Paragon Genomics, Inc., Hayward, CA, USA) on an Illumina MiSeq (Illumina Inc., San Diego, CA, USA). Sequences were analyzed with SOPHiA DDM platform software (SOPHiA Genetics, Lausanne, Switzerland) for lineage assignment. Before being used in this study, all samples underwent an anonymization procedure to comply with the regulations of the local ethics committee (AVR-PPC P09, rev.2; based on Burnett et al.^47^). The collection of human samples was approved by the Institutional Review Board of AUSL Romagna (protocol code “COVdPCR” of 07.02.2020) and informed consent was obtained from all patients. All activities involving the manipulation of infectious virus (virus isolation, viral stock preparation and inactivation assessment) were performed in a Biological Safety Level 3 (BSL-3) facility at the Unit of Microbiology, Greater Romagna Area Hub Laboratory, Cesena, Italy, in compliance with appropriate containment rules. The B.1 and BQ.1.1 variants of SARS-CoV-2 were selected for this study due to their different mutations profile, especially in the Spike protein: B.1 is an early lineage that was widely circulating at the beginning of the pandemic and serves as a representative of the ancestral virus, while, in contrast, BQ.1.1 is a more recent subvariant of Omicron^48–50^. By including both variants, the study aimed to assess the effectiveness of the HHP inactivation against diverse viral strains.

### Preparation of inactivated SARS-CoV-2 by HHP processing and inactivation assessment

Passage three viral stocks were used to infect Vero E6 monolayers at a multiplicity of infection of 0.1 MOI. The supernatant was harvested 72 h post-infection, clarified at 3.500 g for 20 minutes at 4 °C and aliquoted in clear hermetically sealed food-safe polyethylene plastic pouches. High-hydrostatic-pressure mediated inactivation was carried out with a high-pressure system (Avure Technologies Inc., Erlanger, Kentucky, USA). Pouches were positioned directly within the pressure chamber; air was removed from the vessel by an automatic pump and water was pressurized to generate isostatic pressure transmitted through the virus-stock-containing pouches. The pressure inside the chamber was increased at a rate of 200 MPa/minute. During the pressurization phase (which had the purpose of obtaining the inactivation effect), temperature increase inside the chamber was closely monitored in order not to exceed 2–3 °C / 100 MPa; at 600 MPa (the maximum pressure foreseen for the study) the temperature, starting from 3–4 °C, reached a maximum of 20 °C. The pressure was maintained for 5 min. Three different pressures were tested: 400, 500 and 600 MPa. Viral samples were then kept at -80 °C until further analysis. Virus infectivity reduction was assessed by endpoint dilution titration and complete inactivation was tested by culturing the putatively inactivated viral stock on Vero E6 cell culture. Viral replication was evaluated by qRT-PCR. As a control, non-HHP-treated counterparts were titrated and cultured together with HHP-treated viral stocks.

### Antigen purification

Viral stocks were first concentrated by ultracentrifugation at 150.000 g for 1.5 h at 4 °C (Sorvall™ WX+ Ultracentrifuge with rotor fixed-angle F50L-8×39, ThermoFisher, Waltham, Massachusetts, United States). The pellet was resuspended in 2 mL PBS and then purified by ultracentrifugation on a sucrose cushion at 25% w/v in PBS. Tubes were centrifuged at 150.000 g for 2 h at 4 °C. The final pellet was resuspended in PBS at a 50x concentration compared to the initial volume.

### Negative Staining Electron Microscopy (nsEM)

The HHP-treated virus stocks, non-HHP-treated counterparts, and heat-inactivated controls were subjected to negative staining electron microscopy using the Airfuge method^51,52^ at the Unit of Virology of the Istituto Zooprofilattico della Lombardia e dell’Emilia Romagna. Samples were subjected to ultracentrifugation (Airfuge, Beckman Coulter Inc. Life Sciences, Indianapolis, Indiana, USA) for 15 min at 82.000 g. Subsequently, carbon-coated Formvar copper grids were stained with 2% sodium phosphotungstate (pH 6.8) for 1.5 minutes and observed under a Tecnai G2 Spirit Biotwin transmission electron microscope (FEI, Hillsboro, Oregon, USA) at 20.500-43.000x magnification. Images were analyzed qualitatively and quantitively. Morphological differences in viral particles subjected to varying levels of high hydrostatic pressure were evaluated, enabling detailed visualization of structural changes and integrity preservation.

### Western blot

Total proteins of sucrose-purified HHP-processed, non-HHP-processed and heat-inactivated viral stocks were extracted in 20% (v/v) RIPA buffer and quantified by the Bradford method. For each sample, 10 μg of proteins were loaded into NuPAGE 4–12% Bis-Tris Gels. Electrophoretic separation by sodium dodecyl sulfate-polyacrylamide gel electrophoresis (SDS-PAGE) was carried out with NuPAGE MES SDS Running Buffer in reducing conditions at 150 V constant voltage for 30–60 min, depending on the desired target protein molecular weight. Proteins were semi-dry transferred with nitrocellulose Power Blotter Select Transfer Stacks. Blocked blotted membranes (StartingBlock™ Blocking Buffer, 0.05% Tween-20, 1 h at room temperature) were probed with primary anti-SARS-CoV-2 antibodies: Spike Protein S1/S2 Rabbit Polyclonal Antibody (ThermoFisher, PA5-112048), Nucleocapsid Rabbit Monoclonal Antibody (ThermoFisher, MA5-36271) and Membrane Rabbit Polyclonal Antibody (ThermoFisher, SARS-COV2-M-101AP) at a final concentration of 0.5 ug/mL, and Mouse Spike Protein S1 (RBD) Monoclonal Antibody (IZSLER in-house produced, code 3C12) at a concentration of 5 ug/mL. After overnight incubation at 4 °C, membranes were incubated for 1 h at room temperature with HRP (HorseRadish Peroxidase)-conjugated secondary antibodies: Goat anti-Rabbit IgG (H + L) Poly-HRP Secondary Antibody (ThermoFisher, 32260) at 25 ng/mL and Goat anti-Mouse IgG HRP (IZSLER in-house produced, code 72689) at 50 ng/mL. Chemiluminescent signal was detected using SuperSignal^TM^ West Pico PLUS Chemiluminescent Substrate with the iBright 1500FL imaging system with the SmartRange setting, adjusting exposure time if needed. Unless otherwise specified, all reagents and instrumentations were purchased from ThermoFisher. Detailed information on electrophoresis, blotting parameters, and antibody concentrations is summarized in Table S1.

### In vivo immunogenicity testing

The study was conducted on Swiss CD-1 mice under controlled laboratory conditions, adhering to ethical guidelines for animal research. Animal testing was carried out at the animal facility of the Istituto Zooprofilattico Sperimentale della Lombardia e dell’Emilia-Romagna. The study was conducted in accordance with national legal provisions (e.g. DLSG 4/3 2014, n. 26—National implementation of Directive 2010/63/EU). Ethical review was requested and authorization n° 258/2020-PR for animal testing was obtained.

For the study, 28 female adult (6-month-old, weighing ~30–35 grams) were randomly assigned to 7 immunization groups (*n* = 4 per group) to minimize bias. Group allocations included: (1) mice in group 1 were injected with SARS-CoV-2 B.1 HHP-inactivated at 500 MPa in Freund’s complete adjuvant; (2) mice in group 2 were injected with SARS-CoV-2 B.1 HHP-inactivated at 600 MPa in Freund’s complete adjuvant; (3) mice in group 3 were injected with SARS-CoV-2 B.1 heat-inactivated at 65 °C for 1 h in Freund’s complete adjuvant; (4), (5) and (6) mice in group 4, 5 and 6 were injected with SARS-CoV-2 BQ.1.1 HHP-inactivated at 500 MPa, at 600 MPa and heat-inactivated, respectively; (7) mice in group 7 were injected with PBS and Freund’s complete adjuvant (Fig. 2a, b).

For in vivo testing, the purified antigen was diluted to 20 ug/mL (10 ug / 500 uL inoculation volume) in PBS and Freund’s complete adjuvant (Merck KGaA, Darmstadt, Germany). Mice were primed subcutaneously with 10 μg / 500uL of HHP- or heat-inactivated SARS-CoV-2 (or PBS in the control group) with Freund’s complete adjuvant at day 0 and boosted intraperitoneally with the same antigen preparation in PBS (without adjuvant) at day 28. At t53 mice were euthanized by a competent person by exanguination (transcardiac terminal bleeding) under general anesthesia (Ketamine 100 mg/kg + Xylazine 10 mg/kg; intraperitoneal administration), followed by cervical dislocation. All the procedures and activities were performed in compliance with National Legislation (Legislative Decree n. 26/2014) and under Ministry of Health authorisation.

### Enzyme-Linked Immunosorbent Assay (ELISA)

Total anti-SARS-CoV-2 IgG antibodies developed following immunization were measured by indirect ELISA to quantify the total humoral immune response. ELISA was carried out at the Unit of Virology of the Istituto Zooprofilattico della Lombardia e dell’Emilia Romagna. ELISA was performed on days 0, 14, 28, 35 and 53. ELISA plates were coated with purified SARS-CoV-2 antigen (whole virion, beta-propiolactone-inactivated and sucrose-purified; IZSLER, code 31902/46) at a saturating concentration by incubation overnight at 4 °C in ELISA coating buffer (0.05 M carbonate/bicarbonate buffer, pH 9.6). Mouse sera were tested at dilutions of 1:100-1:12.800 and incubated for 1 h at 37 °C. Following washes, an HRP-conjugated goat anti-mouse immunoglobulin antibody (IZSLER, in-house produced, code 72689) was added at a dilution of 1:500 and incubated for 1 h at 37 °C, after which substrate solution (orthophenylenediamine 0.5 mg/ml and 0.02% H_2_O_2_ in 50 mM phosphate citrate buffer, pH 5) was added. The colorimetric reaction was stopped by adding 2 N sulfuric acid; absorbance values were read at 492 nm using an ELISA reader. The endpoint antibody titer was determined via a 4-parameter-logistic (4PL) curve fit analysis of optical density (OD) values for serially diluted sera, with a cut-off value set to three times the background signal. IgG titers were compared within and across groups to monitor immune response maturation at different time-points after immunization and highlight differences among different immunization groups.

### Humoral response specificity characterization

To ascertain which viral proteins elicited the antibody response identified by ELISA for total anti-SARS-CoV-2 IgG, mouse sera were assayed with total viral proteins extracted by chemical methods from whole inactivated SARS-CoV-2 virions. Western blot was performed on days 28 and 53 to evaluate the progression and maturation of the immune response over time.

Total proteins of beta-propiolactone-inactivated and sucrose-purified SARS-CoV-2 (IZSLER, code 31902/46) were extracted in 20% (v/v) RIPA buffer, quantified and loaded into NuPAGE 4–12% Bis-Tris Gels. Proteins were separated by SDS-PAGE at 150 V constant voltage for 60 minutes, semi-dry blotted on a nitrocellulose membrane, and incubated overnight at 4 °C with mouse sera diluted 1:50. Specific mouse anti-SARS-CoV-2 antibodies were detected using HRP-conjugated Goat anti-Mouse IgG (IZSLER in-house produced, code 72689) at a dilution of 1:250. Chemiluminescent signal was detected using SuperSignal^TM^ West Pico PLUS Chemiluminescent Substrate on an iBright FL1500 instrument. Molecular weight of bands corresponding to viral proteins recognized by antibodies in mouse sera was determined through comparison with a molecular weight ladder providing reference points for accurate size estimation (PageRuler™ Plus Prestained Protein Ladder, 10–250 kDa, ThermoFisher). The specificity of the immune response was analyzed by comparing the banding patterns of sera from HHP-immunized mice and heat-immunized mice to those from pre-immune controls or sera from mice immunized with adjuvant alone.

### Virus neutralization test

The neutralizing activity of immunized mice sera was assessed at days 0, 14, 28, 35 and 53. Sera samples were tested at a starting dilution of 1:4 and then diluted 1:2 to 1:2056. Each dilution was then incubated with viral suspension at 1000 TCID_50_/mL at 37 °C for 1 h and transferred to Vero E6 cells. Plates were incubated at 37 °C, 5% CO_2_ for 72 h and then fixed and stained with crystal violet and 4% (v/v) of formaldehyde. Absence or presence of cytopathic effect at each dilution was assessed by comparison of each well with virus control and no-virus control wells. The neutralization titer was defined as the reciprocal of the highest serum dilution capable of inhibiting the appearance of a visible cytopathic effect. To track the immune response’s maturation at various intervals following immunization and to identify variations across immunization groups, neutralization titers were examined both within and between groups.

### ELISpot assay

ELISpot assay was performed 53 days after immunization for the ex vivo quantification of IFNγ-secreting cells after stimulation with an appropriate stimulus in vitro. T-cell response was evaluated on both circulating peripheral blood mononucleate cells (PBMC) and on splenocytes. Whole blood samples were collected from cardiac puncture in lithium-heparin and diluted 1:2 in RPMI 1640 supplemented with 10% heat inactivated FBS, 2 mM L-glutamine, 100 U/mL penicillin, 100 μg/mL streptomycin. PBMC separation was achieved with Ficoll®-Paque Premium (Merck KGaA, Darmstadt, Germany) separation medium at 1000 g for 20 min. Purified PBMC ring was collected and washed twice with supplemented RPMI 1640. Speen were aseptically removed during necropsy and dissociated, then filtered through a 70 μm cell stainer. Splenocytes were washed twice in supplemented RPMI 1640. Both PBMC and splenocytes were diluted to 2.5 × 10^6^ cells/mL. ELISpot assay was carried out with Murine IFNγ ELISpot Kit (Diaclone SAS, Besancon Cedex, France). Cells were seeded (2.5 × 10^5^ cells/well) in skimmed-milk-blocked and anti-IFNγ-capture-antibody coated PVDF (polyvinylidene difluoride) bottomed-well plates. SARS-CoV-2 specific stimulus (beta-propiolactone-inactivated and sucrose-purified SARS-CoV-2 [IZSLER, code 31902/46]) was added at a concentration of 10 μg/mL. PHA (Phytohaemagglutinin) mitogen was used as a positive control at 5 μg/mL; PBS was used as a negative control. Plates were incubated at 37 °C in a 5%-CO_2_-atmosphere enriched chamber for 24 h. For detection, plates were incubated with biotinylated anti-IFNγ Detection Antibody incubated for 2 h at room temperature and Streptavidin-AP (Alkaline Phosphatase) conjugate for 1 h at room temperature. BCIP/NBT substrate (5-bromo-4-chloro-3-indolyl-1-phosphate/nitroblue tetrazolium) was added and incubated until spot development. To quantify the reactogenicity of the sample against the specific SARS-CoV-2 stimulus, the number of blank corrected spots was recorded, and IFNγ production was compared among immunization groups.

### Thermostability assessment

To evaluate the thermostability of HHP-inactivated viral vaccine, the preparation was stored under two distinct temperature conditions, ambient temperature (25 °C) and refrigerated temperature (4 °C) for 7, 14 and 30 days. Antigenicity of the Spike protein was tested through Western blot, as described in paragraph 4.4.2. Positive controls consisted of vaccine samples stored under ideal conditions (-80 °C).

### Statistical analysis

Statistical analysis was carried out with OriginPro 8.5 (OriginLab Corporation, Northampton, MA, USA) using a one-way ANOVA followed by Tukey’s HSD test for pairwise comparisons, provided the normality of data distribution was confirmed via the Shapiro-Wilk test. In cases where the data did not meet normality assumptions, the Kruskal-Wallis test was employed, followed by Dunn’s post-hoc test with Bonferroni correction to account for multiple comparisons. For every statistical test, two levels of statistical significance were considered (**p < 0.05, ***p < 0.01) to assess the results significance.

### Ethics statement

Viral strain isolation from human residual clinical specimens was carried out in compliance with the guidelines of the Declaration of Helsinki, and approved by the Institutional Review Board of AUSL Romagna (protocol code “COVdPCR” of 07.02.2020). Before being used in this study, all samples underwent an anonymization procedure to comply with the regulations of the local ethics committee (AVR-PPC P09, rev.2; based on Burnett et al.^47^). Informed consent was obtained from all patients. In vivo immunogenicity testing on mice was conducted in accordance with national legal provisions (e.g. DLSG 4/3 2014, n. 26—National implementation of Directive 2010/63/EU). Ethical review was requested and authorization n° 258/2020-PR for animal testing was obtained.

## Supplementary information

Supplementary information

## Data Availability

All data supporting the findings of this study are discussed in the main text and summarized in graphs.
