# Supplementary material for: Development and in vivo evaluation of a SARS-CoV-2 inactivated vaccine using high hydrostatic pressure
Source: NPJ Vaccines. 2025 Apr 25;10:83. doi: 10.1038/s41541-025-01136-7 (PMC12032236; doi:10.1038/s41541-025-01136-7)
Supplement: Supplementary file 1 — Supplementary information [file 41541_2025_1136_MOESM1_ESM.pdf]

Table S1. Summary of electrophoresis and blotting parameters, along with antibody probing conditions, commercial or in-house produced primary and secondary antibody and their respective working solution concentrations for the detection of SARS-CoV-2 Spike, Nucleocapsid, and Membrane proteins.

Target		Electrophoresis parameters		Blotting parameters		Primary antibody probing conditions			Secondary antibody probing conditions		
**Protein**	**Molecular weight**	**Voltage (V)**	**Time (minutes)**	**Current (A)**	**Time (minutes)**	**Primary antibody**	**Catalog number**	**Working solution concentration**	**Secondary antibody**	**Catalog number**	**Working solution concentration**
Spike	140-180 kDa (monomer) 540 kDa (trimer)	150	90	1.3	7	SARS-CoV-2 Spike Protein S1/S2 Polyclonal Antibody (rabbit) - ThermoFisher	PA5-112048	0.5 µg/mL	Goat anti-Rabbit IgG (H+L) Poly-HRP Secondary Antibody - ThermoFisher	32260	25 ng/mL
						SARS-CoV-2 2C12 Spike Protein (S1, RBD) – IZSLER in-house produced	3C12	5 µg/mL	Goat anti-Mouse IgG HRP - IZSLER in-house produced	72689	50 ng/mL
Nucleocapsid	~ 46 kDa (monomer) ~ 90 kDa (dimer)	150	45	1.3	7	SARS-CoV-2 Nucleocapsid Monoclonal Antibody (HL448) (rabbit) - ThermoFisher	MA5-36271	0.5 µg/mL	Goat anti-Rabbit IgG (H+L) Poly-HRP Secondary Antibody - ThermoFisher	32260	25 ng/mL
Membrane	25 kDa (monomer) 50 kDa (dimer)	150	30	1.3	7	SARS-CoV-2 Membrane Glycoprotein Polyclonal Antibody (rabbit) - ThermoFisher	SARS-COV2-M-101AP	0.5 µg/mL	Goat anti-Rabbit IgG (H+L) Poly-HRP Secondary Antibody - ThermoFisher	32260	25 ng/mL

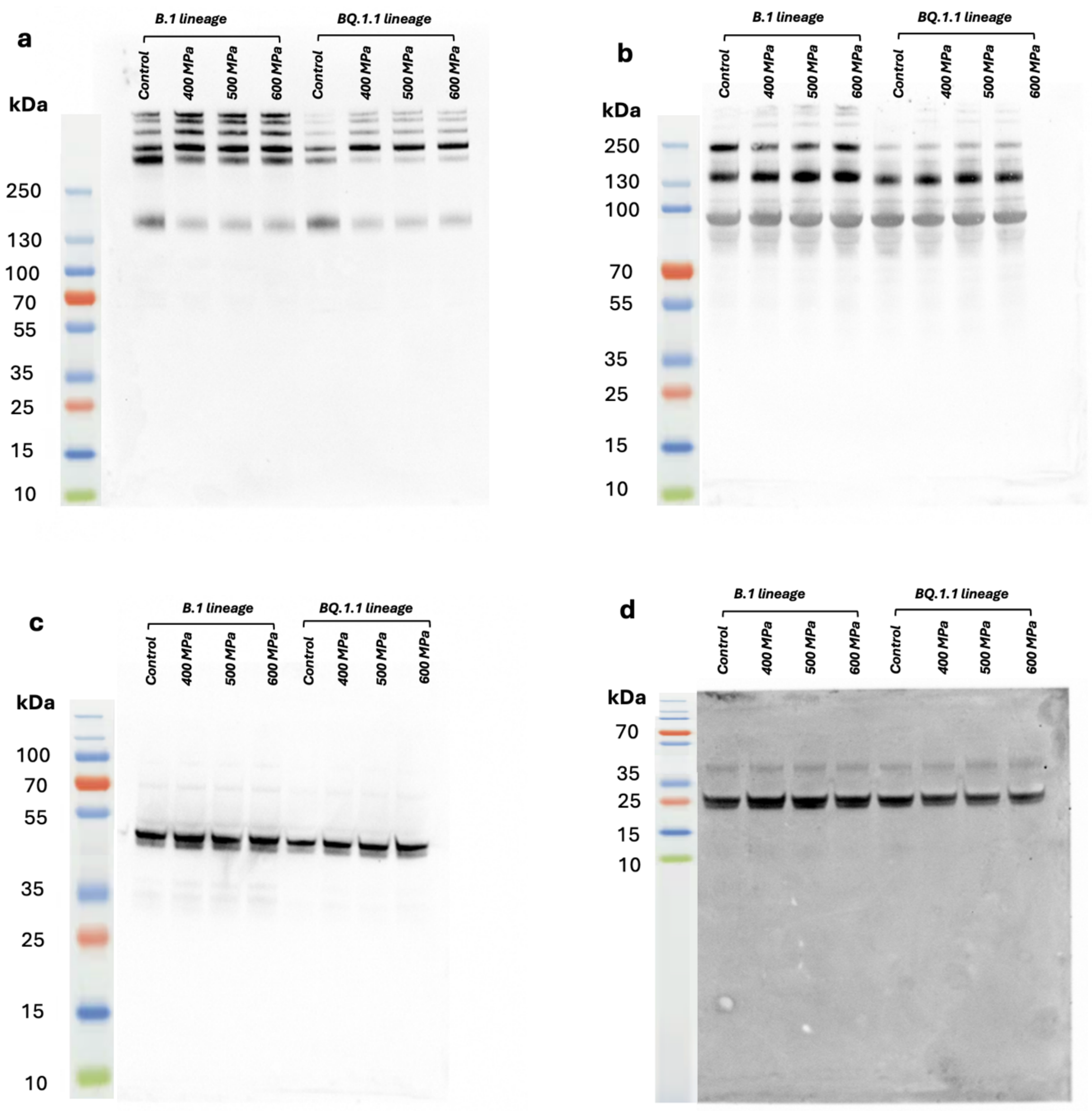

Figure S2. Western blot analysis of B.1 and BQ.1.1 lineages viral suspension subjected to High Hydrostatic Pressure (HHP) inactivation. Results for Spike (panel A [PA5-112048 primary antibody, Thermo Fisher] and B [3C12 primary antibody, IZSLER in-house produced]), Nucleocapsid (panel C [MA5-36271 primary antibody, Thermo Fisher]), and Membrare (panel D [M101-AP primary antibody, Thermo Fisher]) proteins are reported. The molecular weight marker (acquired optically) was juxtaposed with the acquisition by chemiluminescence of the SARS-CoV-2 specific signal for size reference.

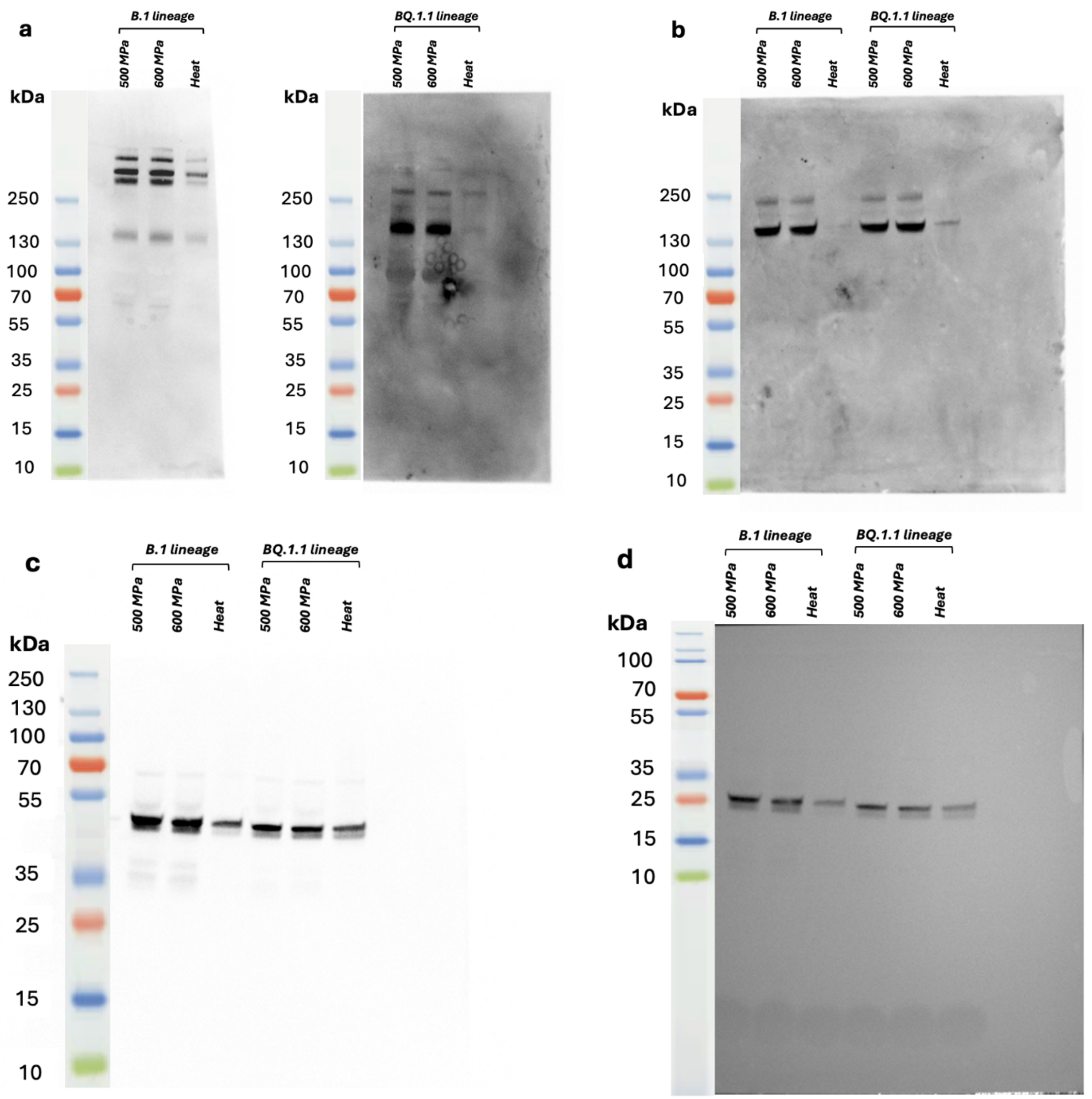

Figure S3. Western blot analysis of B.1 and BQ.1.1 lineages viral suspension inactivated by HHP at 500 and 600 MPa and heat. Results for Spike (panel A [PA5-112048 primary antibody, Thermo Fisher] and B [3C12 primary antibody, IZSLER in-house produced]), Nucleocapsid (panel C [MA5-36271 primary antibody, Thermo Fisher]), and Membrare (panel D [M101-AP primary antibody, Thermo Fisher]) proteins are reported. The molecular weight marker (acquired optically) was juxtaposed with the acquisition by chemiluminescence of the SARS-CoV-2 specific signal for size reference.

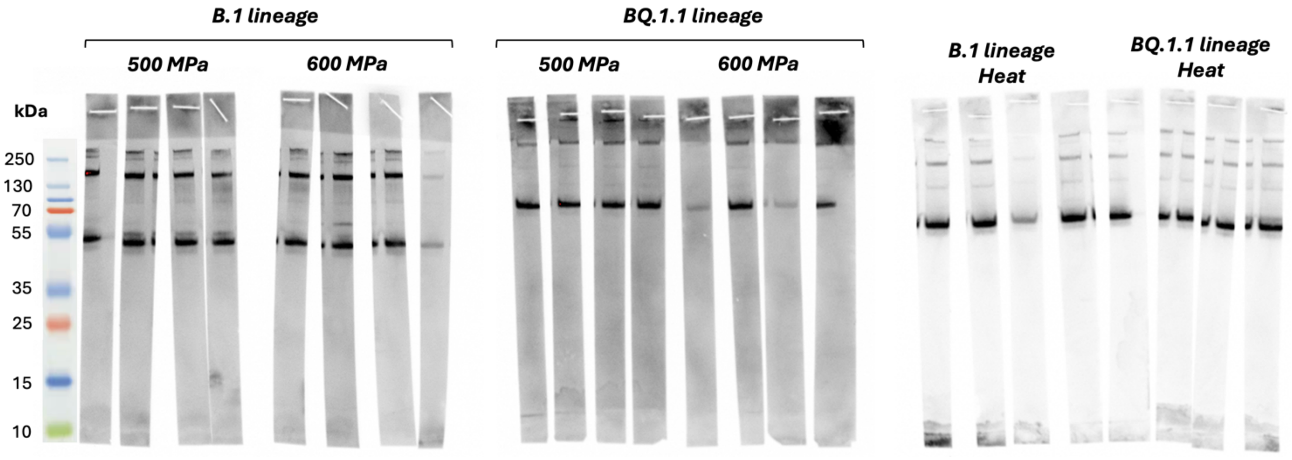

Figure S4. Western blot analysis of sera from mice immunized with B.1 and BQ.1.1 lineage viruses inactivated by 500 MPa, 600 MPa HHP, or heat. The molecular weight marker (acquired optically) was juxtaposed with the acquisition by chemiluminescence of the SARS-CoV-2 specific signal for size reference.

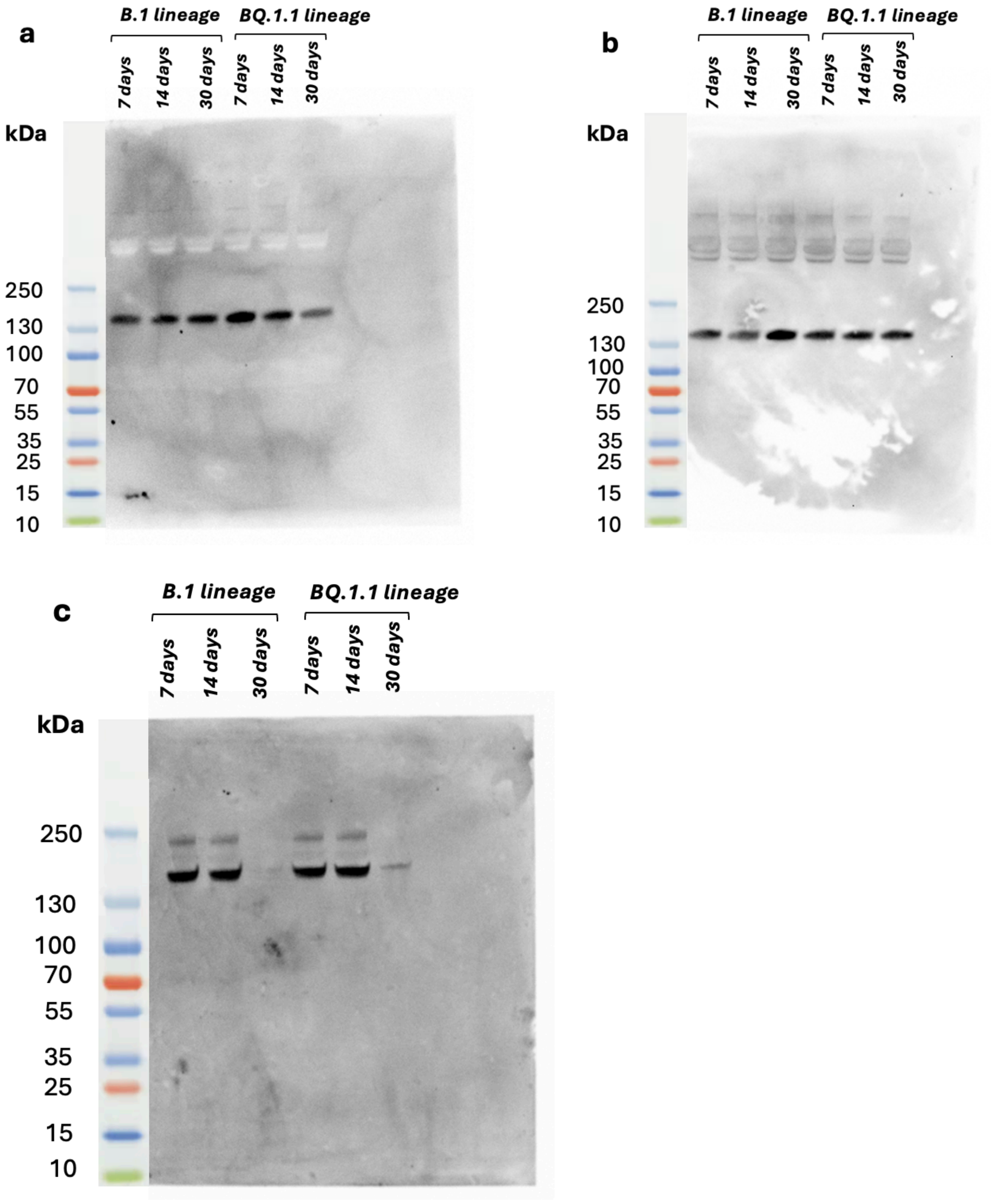

Figure S5. Western blot analysis of the Spike protein signal from SARS-CoV-2 B.1 and BQ.1.1 variants inactivated at 500 MPa, analyzed over various time points (7, 14, and 30 days) upon storage at different temperatures (-80 °C in panel A, 4 °C in panel B, 25 °C in panel C). The molecular weight marker (acquired optically) was juxtaposed with the acquisition by chemiluminescence of the SARS-CoV-2 specific signal for size reference.
